# Enantioselective Protein-Sterol Interactions Mediate Regulation of Both Prokaryotic and Eukaryotic Inward Rectifier K^+^ Channels by Cholesterol

**DOI:** 10.1371/journal.pone.0019393

**Published:** 2011-04-29

**Authors:** Nazzareno D'Avanzo, Krzysztof Hyrc, Decha Enkvetchakul, Douglas F. Covey, Colin G. Nichols

**Affiliations:** 1 Department of Cell Biology and Physiology, and Center for Investigation of Membrane Excitability Diseases, Washington University School of Medicine, St. Louis, Missouri, United States of America; 2 Department of Pharmacological and Physiological Science, Saint Louis University School of Medicine, St. Louis, Missouri, United States of America; 3 Department of Developmental Biology, Washington University School of Medicine, St. Louis, Missouri, United States of America; Biological Research Center of the Hungarian Academy of Sciences, Hungary

## Abstract

Cholesterol is the major sterol component of all mammalian cell plasma membranes and plays a critical role in cell function and growth. Previous studies have shown that cholesterol inhibits inward rectifier K^+^ (Kir) channels, but have not distinguished whether this is due directly to protein-sterol interactions or indirectly to changes in the physical properties of the lipid bilayer. Using purified bacterial and eukaryotic Kir channels reconstituted into liposomes of controlled lipid composition, we demonstrate by ^86^Rb^+^ influx assays that bacterial Kir channels (KirBac1.1 and KirBac3.1) and human Kir2.1 are all inhibited by cholesterol, most likely by locking the channels into prolonged closed states, whereas the enantiomer, *ent-*cholesterol, does not inhibit these channels. These data indicate that cholesterol regulates Kir channels through direct protein-sterol interactions likely taking advantage of an evolutionarily conserved binding pocket.

## Introduction

Cholesterol is the major sterol component of all mammalian cell plasma membranes and plays a critical role in cell function and growth. Excess cholesterol is cytotoxic [1]–[3] resulting in changes in the function of various membrane proteins, including potassium (K^+^) channels [4]–[8], voltage-gated calcium (Ca_v_) channels [9]–[13], voltage-gated sodium (Na_v_) channels [14], [15], and chloride (Cl^−^) channels [16], [17]. Kir channels are expressed in a wide variety of cells including skeletal, cardiac, and vascular myocytes, neurons, and pancreatic β-cells and play numerous key physiological roles including stabilizing the resting membrane potential, regulating K^+^ ion flux across cellular membranes, and regulation of cellular excitability [18]. Previous studies have identified cholesterol suppression of inward rectifier K^+^ channels (“Kir” or “KCNJ” channels) in aortic endothelial cells [8] and by heterologous expression in chinese hamster ovary (CHO) cells [7], [19], [20].

The mechanism by which cholesterol regulates Kir channels has remained elusive since, until recently, experiments could only be done in cell lines where membrane lipid compositions are undefined, difficult to manipulate quantitatively and can vary dramatically from cell to cell and with time. While the reduced inhibitory effect of the C_3_-diastereomer, *epi*-cholesterol, on KirBac1.1 activity is suggestive that cholesterol inhibition may be mediated by direct interactions [8], [21], *epi-*cholesterol and cholesterol orient differently in membranes and may alter membrane properties differently [22], [23]. Thus, changes in Kir channel function due to changes in the physical properties of the lipid bilayer rather than direct binding cannot be excluded based on previous studies.

We have recently shown that *Saccharomyces cerevisiae* can be used to express and purify human Kir channels that can be functionally reconstituted in artificial membranes of defined composition [24]–[26]. In this study, we utilize purified human Kir2.1 channels as well as two bacterial inward rectifiers (KirBac1.1 and KirBac3.1) reconstituted into liposomes with or without cholesterol. With the use of the enantiomeric cholesterol (*ent*-cholesterol)—which has the same effects on membrane properties as natural cholesterol [27]–[29]—we show that in both the prokaryotic and eukaryotic Kir channels the effects of cholesterol are due to direct enantioselective binding of cholesterol to the channel protein, and not an indirect effect due to changes in lipid bilayer properties. Patch-clamp analysis suggests that cholesterol may reduce channel activity by locking the channels into prolonged closed states.

## Methods

### Human Kir2.1 Protein Purification

A Kir2.1-FLAG-His_8_ fusion protein (∼51.5 kDa per monomer) was expressed and purified from the FGY217 strain of *Saccharomyces cerevisiae* as previously described [24]–[26].

### KirBac1.1 Purification

A KirBac1.1-His_6_ fusion protein (∼38.8 kDa per monomer) was expressed and purified from the BL21*(DE3) *E. coli* strain as previously described [30]. Briefly, cells were grown in 1L cultures and induced with 0.5 mM IPTG at OD_600_∼1 for 3 hrs at 37°C. Cells were pelleted and resuspended in 50 mM Tris-HCl pH 8.0, 150 mM KCl, and Complete EDTA-free protease inhibitor tablets (Roche Diagnostics). Cells were lysed by high pressure (20–25 kpsi) homogenization using a Microfluidics Cell Disrupter and solubilized with 30 mM DM for 2 hrs at room temperature. Following centrifugation at 30,000× g for 30 mins, the supernatant was applied to a cobalt affinity column, washed with 20–30 column volumes of wash buffer (50 mM Tris pH 8.0, 150 mM KCl, 10 mM imidazole, and 5 mM DM) and eluted with 1–2 mL of wash buffer containing 500 mM imidazole.

### KirBac3.1 Purification

BL21 CodonPlus RP cells were transformed and grown at 37°C in a shaker to OD_600_∼0.5 then grown at 19°C until an OD_600_∼1.0, followed by induction of KirBac3.1 protein (∼33.7 kDa per monomer) expression with 1 mM IPTG. After 16 hours, cells were harvested by centrifugation, the pellet resuspended in 50 mM Tris-HCl 7.8, 100 mM NaCl, 10 mM KCl , and Complete EDTA-free protease inhibitor tablets (Roche). The resuspended cells were passed through a Parr cell disruptor 5 times, and then centrifuged at 10,000 g for 15 minutes to remove cell debris. Gycerol (26% v/v final) and decylmaltoside (37 mM final) were added to the supernatant and rocked at room tempearture for 3 hours. The mixture was then centrifuged at 10,000 g, for 40 minutes, and the supernatant was mixed with cobalt affinity beads (Talon) for 2 hours. The beads were washed in a disposable plastic column with ∼20 bed volumes of wash buffer (50 mm Tris-HCl, pH 8.0, 100 mm NaCl, 10 mM KCl, 2 mM tridecylmaltoside) and eluted with wash buffer containing 400 mm imidazole.

### Synthesis of Enantiomeric Cholesterol

*ent*-cholesterol, the enantiomer of cholesterol, was synthesized as previously described [31].

### ^86^Rb^+^ Uptake Assay

POPE (1-palmitoyl-2-oleoyl-3-phosphatidylethanolamine) and POPG (1-palmitoyl-2-oleoyl-3-phosphatidylglycerol), cholesterol, and PI(4,5)P_2_ (phosphatidylinositol 4,5-bisphosphate from porcine brain) (Avanti Polar Lipids, Inc.) were solubilized in chloroform, mixed together in the desired concentrations, and dried into a thin film in glass tubes under a nitrogen stream. These lipids were then solubilized in buffer A (450 mM KCl, 10 mM HEPES, 4 mM N-methyl-D-Glucamine, pH 7.5) with 35 mM CHAPS at 10 mg/ml, mixed at the desired mass ratio, and incubated at room temperature for 2 h. Thus, when required, cholesterol was included in the liposomes at the time of formation. Polystyrene columns (Pierce Chemical Co.) were packed with Sephadex G-50 beads, presoaked overnight in buffer A, and spun on a Beckman TJ6 centrifuge until reaching 3,000 rpm. For each sample, 3 µg of protein was added to 100 µl of lipid (1 mg) and incubated for 30 min. Proteo-liposomes were formed by adding the protein/lipid sample to the partially dehydrated columns and centrifuging to 2,500 rpm [24], [25], [32], [33]. The extra-liposomal solution was exchanged by centrifuging the liposomes to 2,500 rpm in partially-dehydrated columns, now containing beads soaked in buffer B (400 mM sorbitol, 10 mM HEPES, 4 mM NMG, pH 7.5). ^86^Rb^+^ uptake was initiated by adding 400 µl of buffer B containing ^86^Rb^+^. At various time points, aliquots of the liposome uptake reaction were flowed through 0.5 ml Dowex cation exchange columns in the NMGH^+^ form to remove extraliposomal ^86^Rb^+^. These aliquots were then mixed with scintillation fluid and counted in a liquid scintillation counter. Valinomycin was used to measure maximal ^86^Rb^+^ uptake. Data were subtracted from uptake counts measured at each time point from protein-free liposomes, and plotted relative to valinomycin-induced uptake counts.

### Electrophysiology of human Kir2.1 in Giant Liposomes

Giant liposomes were prepared in a similar manner as previously described for KirBac1.1 [32]. Briefly, POPE, POPG and PI(4,5)P_2_ were solubilized in K-MOPS buffer (10 mM MOPS acid, pH 7.4, 150 mM KCl) and 35 mM CHAPS and mixed at a 3∶1∶0.04 ratio (a total of 2 mg). Polystyrene columns (Pierce Chemical Co.) were packed with Sephadex G-50 beads, presoaked overnight in K-MOPS buffer, and spun on a Beckman TJ6 centrifuge until reaching 3,000 rpm. Purified Kir2.1 protein was added to the lipids at lipid to protein mass ratio of 33∶1 and incubated at room temperature for 30 min. Proteo-liposomes were formed by adding the protein/lipid sample to the partially-dehydrated columns and spinning to 2,500 rpm, and centrifuged at 100,000 *g* for 1 h at 4°C (TL-100; Beckman Coulter). The liposome pellet was resuspended in 2–3 µl K-MOPS buffer and dried in a desiccator as 2 µl spots on a clean microscope slide for ∼1 h or until completely dried. These spots were then rehydrated with 20 µl K-MOPS buffer overnight at 4°C. Rehydration at room temperature for ∼2 h the next day was sufficient to form giant liposomes.

For patch-clamp, the giant proteo-liposomes were pipetted onto a glass cover slip in an oil-gate chamber [34] and allowed to settle for ∼5 min before starting the solution exchange to wash away debris. Patch-clamp recordings were performed in symmetrical conditions of K-MOPS buffer. Membrane patches were voltage-clamped using a CV-4 headstage, an Axopatch 1-D amplifier, and a Digidata 1322A digitizer board (MDS Analytical Technologies). Patch pipettes were pulled from soda lime glass microhematocrit tubes (Kimble) to a resistance of ∼1–3 MΩ. Single-channel data were digitized at a sampling rate of 10 kHz, and a low-pass analogue filter was set to 1 kHz. Single-channel amplitude histograms were performed using the pClamp 9.2 software suite (MDS Analytical Technologies). The remaining data analysis was performed using Origin7.0 (Microcal).

### Confocal Microscopy

Giant liposomes were formed as above, in the presence or absence of 5% NBD-cholesterol (chemical nomenclature: 22-(N-(7-nitrobenz-2-oxa-1,3-diazol-4-yl)amino)-23,24-bisnor-5-cholen-3β-ol) (Invitrogen). Confocal microscopy was performed using a Zeiss LSM5 Pascal confocal microscope (Zeiss Microimaging, ThornwooD, NY) with a H10×/0.3 Plan-NeoFluar lens (Zeiss). Fluorescence was excited using 488 nm Argon laser line (Lasos, Jena, Germany) and emission was collected using a 505 LP filter (Chroma, Brattleboro, VT). Simultaneously, we collected transmitted light images. Image acquisition and analysis was carried out using Zeiss AIM software.

### Statistical Analysis

Statistical significance was analyzed using an unpaired T-test or one-way ANOVA with Tukey post-hoc analysis as appropriate, and statistical significance (P<0.05) is indicated by an asterisk.

## Results

### Cholesterol Inhibits Purified Prokaryotic and Eukaryotic Inward Rectifier K^+^ Channels

**Figure 1:**
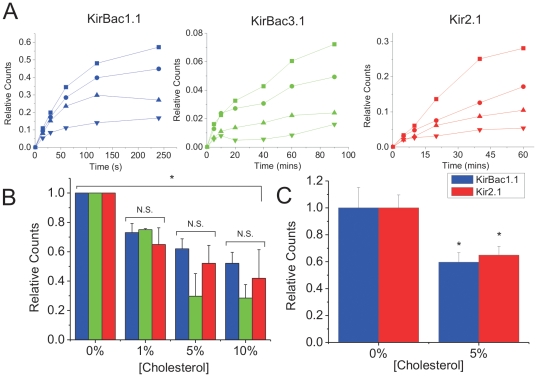
Cholesterol inhibits activity of reconstituted KirBac1.1, KirBac3.1 and human Kir2.1. (**A**) Representative time course of ^86^Rb^+^ uptake into 9∶1 POPE∶POPG liposomes (+1% PI(4,5)P_2_ for Kir2.1) containing increasing amounts of cholesterol reconstituted (0% = ▪; 1% = •; 5% = ▴; 10% = ▾) with KirBac1.1 (left), KirBac3.1 (middle), or Kir2.1 (right) protein, incubated with 450 mM internal and 0 mM external [K^+^]. Uptake was normalized to valinomycin-induced uptake in the same liposomes. (**B**) Channel activity-cholesterol relationship obtained from ^86^Rb^+^ uptake at 240 s for KirBac1.1 (blue; n = 7±s.e.m), at 90 mins for KirBac3.1 (green; n = 6±s.e.m) and at 60 mins for human Kir2.1 (red; n = 6±s.e.m) for each cholesterol concentration. The data are renormalized to uptake in liposomes containing 0% cholesterol. (**C**) ^86^Rb^+^ uptake from KirBac1.1 (blue) and Kir2.1 (red) channels reconstituted into liposomes containing 25% POPG (+1% PI(4,5)P_2_ for Kir2.1) ±5% cholesterol on a POPE background (n = 8 for each). The data were normalized to valinomycin-induced uptake in the same liposomes and re-normalized to uptake in liposomes containing 0% cholesterol. (* P<0.05 assessed by ANOVA).

Bacterial inwardly rectifying K^+^ channels (KirBac1.1 and KirBac3.1) were expressed and purified from *E. coli*, while human Kir2.1 channels were expressed and purified from *S. cerevisiae*. By incorporating these channels into liposomes, previous studies have shown that these proteins form functional K^+^ channels [24]–[26], [30], [32], [35], and that KirBac1.1-mediated ^86^Rb^+^ flux is readily suppressed by the addition of cholesterol to 9∶1 POPE∶POPG liposomes [21]. Here we confirm these results (Fig. 1A and B). In addition, we also show that both KirBac3.1 and human Kir2.1 channel activity are suppressed in liposomes containing as little as 1% cholesterol (mg/mg total lipids) (Fig. 1A and B). Inhibition of channel activity increased in a concentration-dependent manner (Fig. 1B). Notably, cholesterol could not be incorporated into liposomes at concentrations higher than 10%, as indicated by the formation of a heavy precipitate. Interestingly, in this assay, the levels of cholesterol which have an inhibitory effect on Kir channels are well below those typically found in plasma membranes of mammalian cells (between 30–50% of total membrane lipids [2], [3]). However, the degree of inhibition is similar to that previously reported for KirBac1.1 [21]. Importantly, these results show that regulation of Kir channels does not require an intermediary protein that is sensitive to cholesterol.

We previously showed that Kir2.1 channels require anionic phospholipids in addition to PI(4,5)P_2_ for channel activity [24]. Conceivably cholesterol may compete directly or allosterically with POPG to reduce channel activity. Thus, cholesterol inhibition of Kir2.1 and KirBac1.1 channels was also assessed by ^86^Rb^+^ flux in liposomes containing elevated (25%) POPG (Fig. 1C). This does not appear to be the mechanism for cholesterol inhibition, since cholesterol still inhibited KirBac1.1 or Kir2.1 channels in proteo-liposomes containing 25% POPG (Fig. 1C) similarly to channels in proteo-liposomes containing only 10% POPG.

### Cholesterol Does Not Alter Measurable Open Probability or Unitary Conductance in Kir2.1

**Figure 2:**
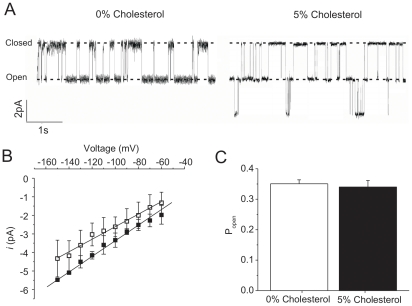
Cholesterol does not affect Kir2.1 open probability or unitary conductance. (**A**) Representative single-channel recordings from human Kir2.1 channels reconstituted into 3∶1 POPE∶POPG giant liposomes at −100 mV in the absence (left) or presence (right) of 5% cholesterol. Neither the unitary conductance (*i*) – voltage (V) relationships (**B**; P = 0.49, n = 8) nor open probability (**C**; P = 0.68, n = 7) were significantly different for human Kir2.1 channels reconstituted into liposomes containing 0% (▪) or 5% (□) cholesterol.

**Figure 3:**
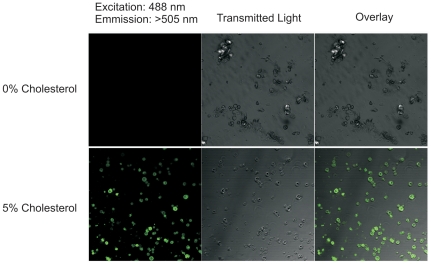
Cholesterol is present in giant liposomes. Giant liposomes were made with 3∶1 POPE∶POPG and 0% or 5% NBD-Cholesterol. Giant liposomes formed without NBD-cholesterol showed no emission above 505 nm following excitation at 488 nm. Robust fluorescence was observed for giant liposomes made in the presence of 5% NBD-cholesterol.

To examine what property of Kir channel function is affected by the presence of cholesterol in the membrane, single-channel recordings were performed by reconstituting human Kir2.1 channels into POPE∶POPG giant liposomes containing 1% PI(4,5)P_2_ and either 0% or 5% cholesterol (Fig. 2A). We find empirically that giant liposomes cannot be formed under the 10% POPG condition used above. Thus, we generated giant liposomes with higher (25%) POPG on a POPE background for these experiments (ie. 25% POPG, 1% PI(4,5)P_2_, 74% POPE or 71% POPE+5% Cholesterol). In the absence of cholesterol, Kir2.1 channels exhibited a unitary conductance of 36.1±1.5 pS and a P_open_ of 0.35±0.01 (n = 8 patches each). In giant liposomes containing 5% cholesterol, no difference was observed in either the measured unitary conductance (33.9±2.9 pS; P = 0.49) (Fig. 2B) or P_open_ (0.34±0.02; P = 0.68) (Fig. 2C) (n = 7). These results cannot explain the inhibitory effect observed under identical conditions in flux experiments data, so to ensure that these results truly reflected single-channel behaviour, and not an inability to incorporate cholesterol into giant liposomes, we made giant liposomes in the presence of a fluorescent analogue of cholesterol (NBD-cholesterol) (Fig. 3). With excitation by 488 nm light, no fluorescence was observed from giant liposomes formed in the absence of cholesterol (Fig. 3 *top*), but robust fluorescence was detected from liposomes containing 5% NBD-cholesterol (Fig. 3 *bottom*), indicating that the patch-clamp experiments are not limited by the ability to incorporate cholesterol into giant liposomes.

Thus, our electrophysiological results do in fact reflect Kir2.1 single-channel behaviour, and support the idea that cholesterol may act to cause long-term “silencing” of channels by inducing prolonged closed states. One consequence of such a mechanism would be a reduction in the “observed” average number of channels in each patch with the inclusion of cholesterol in the membranes. Although not statistically significant, this appears to be the trend: we detected 6.7±1.8 channels per active patch (range = 1–14 channels/patch) in the absence of cholesterol, versus 4.2±1.8 channels per patch (1–10 channels/active patch) in the presence of 5% cholesterol (P = 0.29; n = 7 and n = 6 respectively). Power analysis indicates that for 80% power, data from more than 85 patches would have to be collected to determine if this trend is significant. Given the cost of protein purifications, and the variability of the patches, we have not attempted to perform this number of experiments.

### Enantiospecific Binding of Cholesterol to Prokaryotic and Eukaryotic Inward Rectifier K^+^ Channels

**Figure 4:**
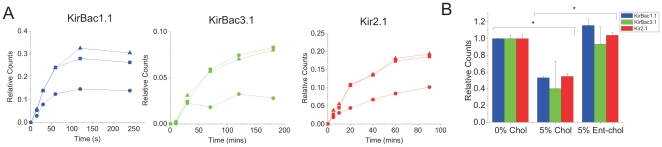
Cholesterol enantiospecifically binds prokaryotic and eukaryotic Kir channels. (**A**) Representative time course of ^86^Rb^+^ uptake into 9∶1 POPE∶POPG liposomes (+1% PI(4,5)P_2_ for Kir2.1) containing 0% cholesterol (▪), 5% cholesterol (•) or 5% *ent-*cholesterol (▴) reconstituted with KirBac1.1 (left), KirBac3.1 (middle), or Kir2.1 (right) protein, with 450 mM internal and 0 mM external [K^+^]. (**B**) KirBac1.1 (blue), KirBac3.1 (green) and human Kir2.1 (red) channel activity-cholesterol relationship obtained from ^86^Rb^+^ uptake counts. The data are renormalized to uptake in liposomes containing 0% cholesterol. (n = 6 for each protein, * P<0.05 assessed by ANOVA).

Cholesterol can act to alter the function of membrane proteins either through direct binding or indirectly through changes in membrane fluidity, stiffness, thickness, permeability and/or phospholipid packing [2], [3], [36]–[41]. To distinguish between these possible mechanisms for cholesterol effects on Kir channel function we utilized the enantiomer of cholesterol (*ent*-cholesterol), which has the same effects on membrane properties as cholesterol [27], [28], [42], yet cannot interact identically with the anisotropic structure of proteins. By comparing the effect of either 5% cholesterol or *ent*-cholesterol on ^86^Rb^+^ uptake we show that the effect of cholesterol on KirBac1.1, KirBac3.1 and human Kir2.1 channel activity is enantioselective (Fig. 4A and B). This differential effect of cholesterol and *ent*-cholesterol on prokaryotic and eukaryotic inward rectifier K^+^ channels implies that in these channels, cholesterol inhibition depends on enantiospecific protein-sterol interactions, rather than on indirect changes of the physical properties of the lipid bilayer.

## Discussion

A growing number of ion channels have been shown to be regulated by components of the lipid membranes [4]–[8], [10], [11], [13], [19]–[21], and previous studies have indicated that cholesterol can inhibit both mammalian and bacterial Kir channel currents [7], [19], [20]. Here, we have shown that in POPE/POPG liposomes, containing prokaryotic KirBac1.1, KirBac3.1 or human Kir2.1 are similarly modulated by as little as 1% cholesterol (mg/mg total lipids). This is in stark contrast to the opposing effects of PI(4,5)P_2_ on prokaryotic and eukaryotic Kirs [25], [35]. Interestingly, the levels of cholesterol necessary to regulate Kir2.1 channels are well below the levels reported in plasma membranes of mammalian cells, which range between 30–50% of total membrane lipids [2], [3].

Effects of sterols on lipid membrane properties generally show no entantioselectivity. In particular, no enantioselective effects on phase-transition properties [42] and lipid packing in monolayers, and on bilayer properties as measured by calorimetrey, X-ray diffraction, and neutron density measurements have been reported between cholesterol and *ent*-cholesterol [27]. On the other hand, studies with enzymes and transporters that use cholesterol as a substrate demonstrate a high entantiomer selectivity of interaction [43], [44], while lipid domain forming peptides have also been shown to have differential interactions with cholesterol entantiomers [42]. Thus, the differential actions of cholesterol versus *ent-*cholesterol on protein function reflect the existence of direct protein-sterol interactions [27]–[29].

Previous studies have attempted to address whether the regulation of Kir channel function results from direct channel-sterol interactions or indirect effects such as changes to the physical properties of the lipid bilayer or regulation by an intermediary cholesterol-sensitive protein through the use of the C_3_-diastereomer, *epi*-cholesterol [8], [21]. *Epi*-cholesterol inhibited KirBac1.1 channels less than the equivalent amount of cholesterol [8], [21]. Partial substitution of cholesterol with *epi*-cholesterol in mammalian cells resulted in an increase in current relative to control, which may suggest specific protein-sterol interactions [8], [21]. However, the interpretation of such studies is not trivial since (a) mediation through accessory proteins cannot be excluded from recordings done in eukaryotic cell membranes and (b) *epi-* and natural cholesterol orient differently in membranes, and can thus alter membrane properties differently [22], [23]. No enantioselectivity has been observed for cholesterol effects on various lipid bilayer and monolayer properties [27], [28]. Thus, determining the enantioselectivity of purified channel regulation by cholesterol with the use of *ent-*cholesterol is a more suitable approach. This assay has been used to determine the enantioselectivity of cholesterol regulation of numerous channels and receptors (for complete list see review by Covey, 2009 [45]), including a recent study of calcium-activated BK channels [46]. Using this assay, we observed that both prokaryotic and eukaryotic Kir channels are indeed enantioselective for cholesterol, indicating that modulation by this sterol is mediated by direct binding to the channel. Since the degree of channel modulation by cholesterol is similar between prokaryotic and eukaryotic Kir channels and since cholesterol is not present in prokaryotic membranes [47], we speculate that modulation of eukaryotic Kirs by cholesterol utilizes a conserved “binding pocket” also found in prokaryotic Kirs. It has been previously suggested that cholesterol may bind to Kir channels in a confirmation that requires the cytoplasmic domain to be close to the membrane [48] and that this confirmation may be favored when residue 222 in Kir2.1 channels is a leucine, and unfavourable when isoleucine [48]. Although KirBac channels do not have leucine at the equivalent position, comparison of the eukaryotic and bacterial channel structures indicates that the cytoplasmic domains of KirBac channels are already closer to the bilayer than eukaryotic Kir channels [49] and may be already close enough to interact with cholesterol.

Unitary conductances and open probabilities were not significantly different for human Kir2.1 in the presence or absence of cholesterol in liposomes containing 25% POPG. The presence of cholesterol in giant liposomes under these conditions was verified using fluorescent NBD-cholesterol (Fig. 3). Similarly, despite decreases in whole-cell currents, no changes in single-channel properties were reported for either I_K1_ in bovine aortic endothelial cells or for Kir2.1 currents over-expressed in CHO cells treated with MβCD-cholesterol [7], [8]. We show that increasing POPG concentration does not alter cholesterol inhibition of Kir2.1 or KirBac1.1 channels (Fig. 1C), and thus cannot account for the discrepancy between flux experiments where cholesterol reduced channel activity by ∼45% (Fig. 1) and our patch clamp experiments on giant liposomes where no effect on channel conductance or P_open_ were observed (Fig. 2). Together with our observation that cholesterol binds directly to Kir channels, these data support the idea that when bound, cholesterol induces conformational changes that lead to a prolonged closed state that cannot be detected by single-channel analysis [7]. Consequently, we are only able to detect single-channel currents from proteins not interacting with cholesterol.
